# PRP4 Promotes Skin Cancer by Inhibiting Production of Melanin, Blocking Influx of Extracellular Calcium, and Remodeling Cell Actin Cytoskeleton

**DOI:** 10.3390/ijms22136992

**Published:** 2021-06-29

**Authors:** Muhammad Bilal Ahmed, Salman Ul Islam, Young Sup Lee

**Affiliations:** BK21 FOUR KNU Creative Bioresearch Group, School of Life Sciences, Kyungpook National University, Daegu 41566, Korea; muhammad786@knu.ac.kr (M.B.A.); salman2013@knu.ac.kr (S.U.I.)

**Keywords:** PRP4, melanocyte, cAMP, calcium-sensing receptor, actin cytoskeleton, drug resistance

## Abstract

Pre-mRNA processing factor 4B (PRP4) has previously been shown to induce epithelial-mesenchymal transition (EMT) and drug resistance in cancer cell lines. As melanin plays an important photoprotective role in the risk of sun-induced skin cancers, we have investigated whether PRP4 can induce drug resistance and regulate melanin biosynthesis in a murine melanoma (B16F10) cell line. Cells were incubated with a crucial melanogenesis stimulator, alpha-melanocyte-stimulating hormone, followed by transfection with PRP4. This resulted in the inhibition of the production of melanin via the downregulation of adenylyl cyclase-cyclic adenosine 3′,5′-monophosphate (AC)–(cAMP)–tyrosinase synthesis signaling pathway. Inhibition of melanin production by PRP4 leads to the promotion of carcinogenesis and induced drug resistance in B16F10 cells. Additionally, PRP4 overexpression upregulated the expression of β-arrestin 1 and desensitized the extracellular calcium-sensing receptor (CaSR), which in turn, inhibited the influx of extracellular Ca^2+^ ions. The decreased influx of Ca^2+^ was confirmed by a decreased expression level of calmodulin. We have demonstrated that transient receptor potential cation channel subfamily C member 1 was involved in the influx of CaSR-induced Ca^2+^ via a decreasing level of its expression. Furthermore, PRP4 overexpression downregulated the expression of AC, decreased the synthesis of cAMP, and modulated the actin cytoskeleton by inhibiting the expression of Ras homolog family member A (RhoA). Our investigation suggests that PRP4 inhibits the production of melanin in B16F10 cells, blocks the influx of Ca^2+^ through desensitization of CaSR, and modulates the actin cytoskeleton through downregulating the AC–cAMP pathway; taken together, these observations collectively lead to the promotion of skin carcinogenesis.

## 1. Introduction

The melanoma cell line B16F10 can produce melanin and displays metastatic behaviors. These cells are widely used to study melanogenesis and depigmentation [1], tumor metastasis [2], and cytotoxicity measurements of various substances in skin models [3]. Melanogenesis is a complex process regulated by enzymatic cascades, including tyrosinase, tyrosinase-related protein (TRP)-1, and their transcription factors like microphthalmia-associated transcription factor (MITF), cAMP response element-binding protein (CREB), and extracellular-regulated kinase (ERK) [4]. The most crucial hormone in stimulating melanogenesis is alpha-melanocyte stimulating hormone (α-MSH), which binds and induces MITF. α-MSH binds to melanocortin 1 receptors, which produces cAMP, and cAMP phosphorylates the CREB transcription factor, which in turn promotes MITF activation. MITF binds to the promoter regions of melanin production genes and positively regulates their transcription of TRP-1 and tyrosinase [5,6,7]. As previously identified, melanin has a photoprotective role and decreases the risk of sun-induced skin cancers [8,9]. It is also reported to serve as a physical barrier that scatters ultraviolet radiations, and as an absorbent filter that reduces the penetration of these radiations through the epidermis [10]. Melanosomes in dark skin show resistance to lysosomal degradation and remain intact throughout the epidermal layers. Moreover, they form supranuclear caps in keratinocytes and melanocytes, and protect against ultraviolet radiations-induced damage [11,12].

Extracellular calcium-sensing receptor (CaSR) is a G-protein-coupled receptor (GPCR) predominantly expressed in the kidneys and parathyroids, where it regulates the secretion of parathyroid hormone and renal tubular calcium reabsorption [13]. The expression of CaSR has also been reported in other tissues including lung, heart, skin, brain, pancreas, intestine, bone, bone marrow, lens epithelium, and thyroid, where its role remains yet to be defined [14]. When a ligand binds to CaSR, it stimulates the release of Ca^2+^ from intracellular stores via activation and accumulation of phospholipase C and inositol 1,4,5–trisphosphate, respectively, followed by an influx of Ca^2+^ [14].

Previous studies have demonstrated that CaSR, which is expressed on the surface, may undergo endocytosis, which is initiated with phosphorylation by protein kinase C or G protein-coupled receptor kinase (GRKs) [15,16]. This process involves β-arrestins (ARRBs) and is facilitated by Ras-related protein Rab-7a, Ras-related protein Rab-11A, and adaptor protein-2 [17,18,19]. CaSR is translocated to the lysosomes for degradation after endocytosis or is recycled to the cell membrane, which contributes to the receptor resensitization [20,21]. ARRBs are ubiquitously expressed proteins that play a crucial role in the desensitization and internalization of most GPCRs [22]. Min Pi et al. analyzed luciferase activity in HEK293 cells that were cotransfected with rat CaSR, and an SRE-luciferase reporter constructs in the presence or absence of ARRB1 and ARRB2. They reported that ARRB isoforms downregulate the level of CaSR-induced luciferase activity [16]. Also, interaction between ARRB1 and the CaSR has been shown in mammalian cells by coimmunoprecipitation experiments [16]. It has also been observed that overexpression of both ARRB1 and ARRB2 will have a negative influence on CaSR-mediated inositol phosphate production in GripTite293 cells [15].

Ca^2+^ influx through store-operated channels has been shown to be induced by CaSR, which, in many cases, has been identified as the transient receptor potential (TRP) family members [23]. Yassine El Hiani et al. reported that ERK and transient receptor potential canonical 1 (TRPC1) were required for CaSR-stimulated MCF-7 breast cancer cell proliferation [24]. In endothelial cells, it is probable that TRPC1 is a contributor to the formation of store operated Ca^2+^ channels [25].

Cyclic adenosine 3′,5′-monophosphate (cAMP) mediates diverse effects in cytoskeletal dynamics, cell migration, and cell adhesion [26,27]. Elevation of cAMP and activation of PKA have been reported to be involved in microfilament assembly [28], mammary epithelial cell migration on laminin [29], formation of filopodia and lamellipodia in response to follicle-stimulating hormone [30], and activation of Cdc42 and Rac [31,32]. It has been suggested that inhibition of Rho is involved in mediating the effects of cAMP and PKA on actin-based movement and morphology [33,34,35].

The protein, PRP4, was first identified from screens designed to isolate genes essential for pre-mRNA splicing processes in *Schizosaccharomyces pombe*, which reported that mutations of fission yeast PRP4 kinase caused the accumulation of pre-mRNA species [36]. Human *PRP4* encodes a 1007-amino acid protein containing an N-terminal 340-amino acid Arg/Ser-rich domain, which is commonly found in pre-mRNA splicing factors [37]. It has been reported that in addition to RS domain, PRP4 contains a kinase domain, which shares homology with cyclin-dependent kinases and mitogen-activated protein kinases [38,39]. It is noteworthy that kinase domain is absent in other pre-mRNA splicing factors. Mutations in PRP4 result in pre-mRNA accumulation and in an impaired G1/S transition during the cell cycle [40]. Studies on PRP4 have demonstrated its diverse effects on kinases, transcription factors, chromatin remodeling factors, spindle checkpoint proteins, and cancer cell growth [37,41,42,43]. In our previous study, we have shown that PRP4 is involved in the promotion of drug resistance in cancer cell lines by inducing changes to the cell cytoskeletal architecture and therefore induce epithelial–mesenchymal transition (EMT) [44]. In this current study, we have investigated the regulatory effect of PRP4 on melanin production in B16F10 cells and observed that PRP4 overexpression inhibited the production of melanin. In addition, PRP4 increased the ARRB1-induced desensitization of CaSR, which subsequently decreased the influx of Ca^2+^ and downregulated the activity of calmodulin. Furthermore, cAMP signaling was inhibited by PRP4 overexpression altering the morphology of B16F10. Our investigation suggests that PRP4 has an important role in regulating the morphological and migratory characteristics of B16F10 cells and may lead to the promotion of skin melanoma through the reduction of melanin production.

## 2. Results

### 2.1. PRP4 Inhibits the Production of Melanin in B16F10 Cells and Promotes Skin Cancer

**Figure 1:**
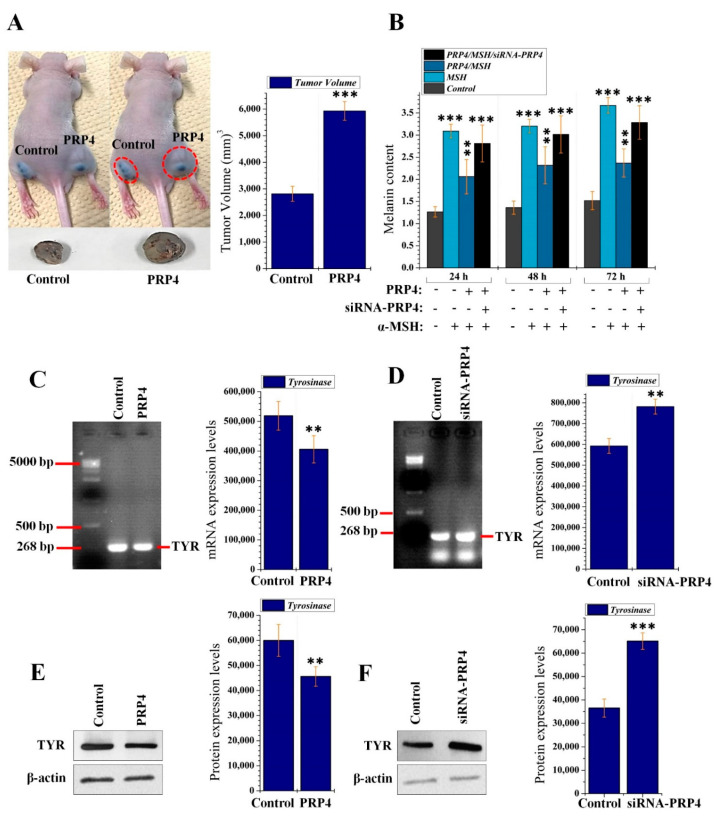
PRP4 inhibits the production of melanin in B16F10 cells, which subsequently leads to the promotion of skin cancer. (**A**) Nude mice (*n* = 6) received parental and PRP4-transfected B16F10 cells into the left and right flank, respectively, by subcutaneous injection. Data were collected from 3 independent experiments. *** *p* < 0.001. For clear visibility of the tumors in vivo, the same mouse was photographed twice; the tumors are encircled in the latter set of photographs. (**B**) Melanin production in PRP4 transfected B16F10 melanoma cells. The cells were transfected with 5 μg PRP4 for 24, 48, and 72 h, and α-MSH was used as a positive control. Data were collected from 3 independent experiments. ** *p* < 0.01, *** *p* < 0.001. (**C,D**) Triplicate total RNA samples from control and the PRP4 and siRNA-PRP4 transfected cells were analyzed by RT-PCR. ** *p* < 0.01. (**E**,**F**) Solubilized proteins (20 μg) from PRP4 and siRNA-PRP4 transfected cultured cells were subjected to Western blot analysis using the corresponding antibodies for detection of respective TYR protein. The blot was simultaneously incubated with an antiproteins antibody to show that each electrophoretic lane was loaded with the same amount of protein. β-actin was used as a standard. Data are presented as mean ± standard deviation (SD) of at least three independent experiments (*n* = 3). ** *p* < 0.01, *** *p* < 0.001. Treatment or transfection related abbreviations: PRP4 = PRP4 cDNA plasmid transfection; siRNA-PRP4 = transfection of siRNA-PRP4; α-MSH = 100 nm treatment of α-MSH; TYR = tyrosine.

In our previous investigations, we conducted in vitro studies and reported that PRP4 participates in the development of drug resistance in cancer cell lines [44,45]. In the current investigation, we analyzed the role of PRP4 in vivo using a subcutaneous xenotransplant tumor model by injecting B16F10 cells into BALB/c-n mice. Briefly, parental B16F10 cells are injected on the left, while PRP4-transfected B16F10 cells are injected on the right (according to the left and right of the photo in Figure 1A). Tumor sizes were regularly measured at 3- or 4-day intervals until 50 days post implantation. PRP4-transfected B16F10 cells show enhanced tumor growth relative to parental B16F10 cells, and body weight (average weight = 22.5 g) was not affected (Figure 1A). Tumor volume was measured according to the formula described in Materials and Methods and presented in a bar graph. We were curious to find the mechanism through which PRP4 promoted skin cancer, therefore, the effect of PRP4 on the melanin content of B16F10 cells was also investigated in this study. The α-MSH binding to melanocortin 1 receptor (MC1R) activates the AC that increases the cAMP levels, which subsequently activates MITF [46,47]. MITF binds to the promoter regions of melanin production genes, TRP-1 and tyrosinase, and positively regulates their transcription [5,6,7]. In this study, to begin we investigated the PRP4 regulatory effect on the production of melanin in B16F10 cells by treating them with α-MSH, followed by PRP4/siRNA-PRP4 transfection. We observed that PRP4 overexpression blocked the α-MSH-induced melanin production in B16F10 cells, whereas siRNA-PRP4 reversed the inhibitory effect of PRP4 (Figure 1B). We further analyzed the mRNA and protein levels of tyrosine, which were both downregulated by the overexpression of PRP4 (Figure 1C–F). Our data indicate that PRP4 may decrease the production of melanin in B16F10 cells by downregulating AC–cAMP–MITF–tyrosinase signaling pathway, which subsequently leads to the promotion of skin cancer.

### 2.2. PRP4 Regulates ARRB1-CaSR Pathway

**Figure 2:**
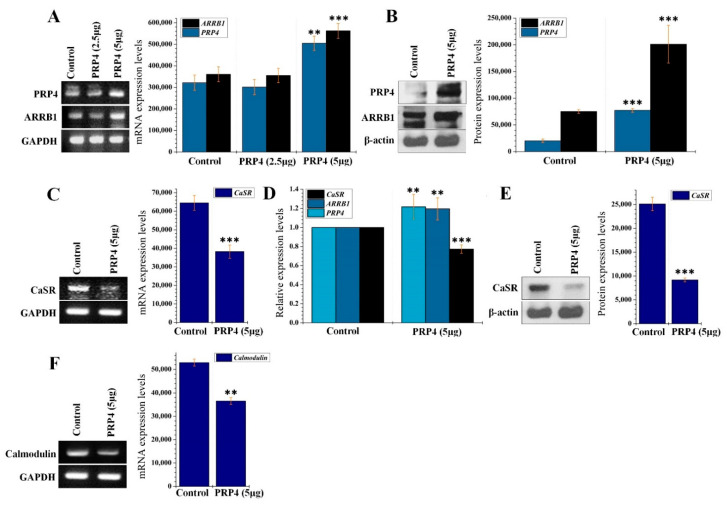
PRP4 regulates ARRB1–CaSR pathway. (**A**) Triplicate total RNA samples from control and the PRP4-transfected were analyzed by RT-PCR. *** *p* < 0.001. (**B**) Solubilized proteins (20 μg) from PRP4 transfected growing cells were subjected to Western blot analysis using the corresponding antibodies for detection of respective proteins. The blot was simultaneously incubated with an antiproteins antibody to show that each electrophoretic lane was loaded with the same amount of protein. β-actin was used as a standard. *** *p* < 0.001. (**C**) Triplicate total RNA samples from control and the PRP4-transfected were analyzed for CaSR by RT-PCR. *** *p* < 0.001. (**D**) Gene expressions were quantified by using quantitative Real-Time PCR (qPCR). ** *p* < 0.01, *** *p* < 0.001. (**E**) Solubilized proteins (20 μg) from PRP4 transfected growing cells were subjected to Western blot analysis using the corresponding antibodies for detection of respective proteins. The blot was simultaneously incubated with an anti-proteins antibody to show that each electrophoretic lane was loaded with the same amount of protein. β-actin was used as a standard. *** *p* < 0.001. (**F**) Triplicate total RNA samples from control and the PRP4-transfected were analyzed for calmodulin by RT-PCR. GAPDH and β-actin were used as a standard. Data shown are mean with 95% confidence intervals of three qPCR assays. Data are presented as mean ± standard deviation (SD) of at least three independent experiments. ** *p* < 0.01.

PRP4 was overexpressed in B16F10 cells by transfecting cells with PRP4 expressing plasmid at 2.5 µg and 5 µg concentration (Figure 2A,B); the latter produced the optimum expression (1.31-fold and 3.80-fold increase at mRNA and protein levels, respectively) and was selected for continuing experiments. We analyzed the effect of PRP4 overexpression upon ARRB1 expression. PRP4 overexpression significantly increased ARRB1 (1.55-fold) at the mRNA level (Figure 2A) and increased the protein level of ARRB1 (2.67-fold) as detected by Western blot (Figure 2B). Previously, it has been shown that ARRB1 induces the desensitization of CaSR, which is continuously exposed to Ca^2+^, and yet remains very sensitive to even a small change in serum calcium. Desensitization of CaSR may be an important control point for regulating CaSR signaling [16]. To link the ARRB1-CaSR pathway with PRP4, we conducted RT-PCR as well as qPCR, which demonstrated that PRP4 induced the desensitization of CaSR by upregulating ARRB1 (Figure 2C,D). Similar findings were observed through Western blotting (Figure 2E). We also analyzed the mRNA levels of calmodulin, which were interestingly downregulated by PRP4 (Figure 2F).

**Figure 3:**
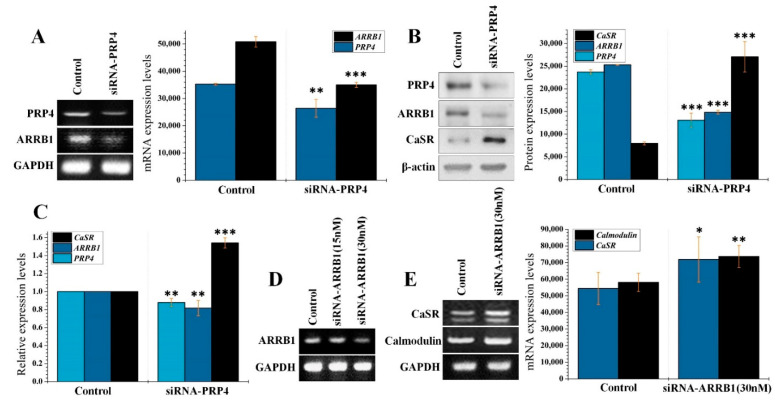
Interaction between PRP4 with the ARRB1–CaSR pathway. (**A**) Gene expression was assessed using siRNA-PRP4. Cells were assayed in triplicate from autonomous batches of RNA and sequestered from frozen pellets of cells grown under the same conditions on different days. The data shown are the mean and 95% confidence intervals of three RT and qPCR assays. GAPDH was used as the standard control. ** *p* < 0.01, *** *p* < 0.001. (**B**) Solubilized proteins (20 μg) from siRNA-PRP4-transfected cultured cells were subjected to Western blot analysis using the corresponding antibodies for detection of respective proteins. The blot was simultaneously incubated with an antiproteins antibody to show that each electrophoretic lane was loaded with the same amount of protein. *** *p* < 0.001. (**C**) Gene expressions were quantified using quantitative Real-Time PCR (qPCR) in siRNA-PRP4 transfected cells. ** *p* < 0.01, *** *p* < 0.001. (**D,E**) Triplicate total RNA samples from control and the siRNA-ARRB1 transfected cells were analyzed by RT-PCR. GAPDH was used as a standard. Data are presented as mean ± standard deviation (SD) of at least three independent experiments. * *p* < 0.05, ** *p* < 0.01. Treatment or transfection related abbreviations: siRNA-ARRB1 = transfection of siRNA-ARRB1.

To confirm that ARRB1 is upregulated by PRP4 overexpression, we performed PRP4′s siRNA-mediated knockdown using a pool of three target-specific 19–25 nucleotide-long siRNAs (siRNA-PRP4). The siRNA-PRP4 inhibited both the PRP4 and the PRP4-induced expression of ARRB1 (Figure 3A). We further confirmed these results through Western blot and qPCR (Figure 3B,C). Additionally, we optimized the conditions for siRNA-ARRB1 transfection, where 30 nM concentration significantly blocked the expression of ARRB1 (Figure 3D). Furthermore, siRNA-ARRB1 transfection activated the expression of CaSR and calmodulin, which indicates that these genes are the targets of ARRB1 (Figure 3E). Collectively, these findings indicate that PRP4 overexpression activates the expression ARRB1, which, in turn, induces the desensitization of CaSR and subsequently suppresses the expression of calmodulin.

### 2.3. PRP4 Overexpression Reduces the Influx of Ca^2+^ and Downregulates Calmodulin

**Figure 4:**
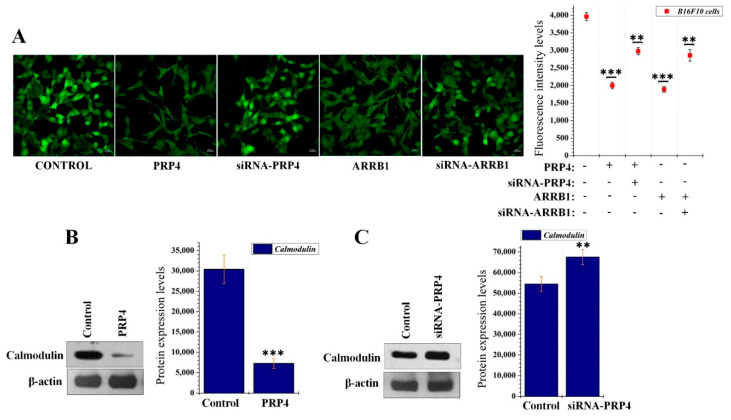
PRP4 reduces the influx of Ca^2+^ and decreases the expression of calmodulin. (**A**) PRP4, quadrant 4. ARRB1, or siRNA-ARRB1-transfected B16F10 cells were stained with Cal-520 AM to observe the intracellular calcium ions levels through fluorescence intensity levels. **p < 0.01, *** *p* < 0.001. (**B**,**C**) PRP4 and siRNA-PRP4-transfected B16F10 cells were analyzed with Western blotting to observe calmodulin (CALM1) expression. Data are presented as mean ± standard deviation (SD) of at least three independent experiments. ** *p* < 0.01, *** *p* < 0.001. Treatment or transfection related abbreviations: ARRB1= transfection of ARRB1 plasmid.

We hypothesized that the influx of Ca^2+^ may be blocked through PRP4 and ARRB1-mediated desensitization of CaSR. Therefore, we cultured B16F10 cells in Ca^2+^-rich media (supplemented with 1 mM calcium) followed by transfection with either PRP4, ARRB1, siRNA-PRP4, or siRNA-ARRB1. Intracellular Ca^2+^ levels were evaluated and were reduced by PRP4 overexpression, whereas siRNA-mediated inhibition of PRP4 restored the intracellular concentration of Ca^2+^. Consistent results were obtained with ARRB1 and si-ARRB1 transfection (Figure 4A). To confirm our hypothesis that PRP4-induced inhibition of intracellular Ca^2+^ would downregulate the protein expression of calmodulin, we conducted Western blot, which demonstrated that PRP4 overexpression decreased the expression of calmodulin (Figure 4B). Furthermore, siRNA-PRP4 transfection restored calmodulin protein expression (Figure 4C). These results indicated that PRP4 overexpression may inhibit the influx of Ca^2+^ and downregulate the calmodulin through enhancing the ARRB1-mediated desensitization of CaSR.

### 2.4. PRP4-Regulated CaSR-Induced Ca^2+^ Influx Occurs through the TRPC1 Channel in B16F10 Cells

**Figure 5:**
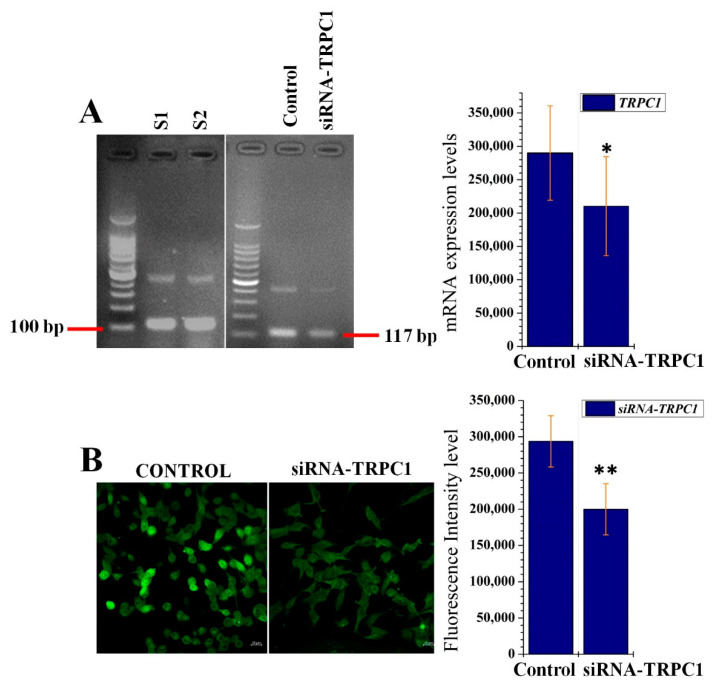
PRP4-regulated CaSR-induced Ca^2+^ influx occurs through the TRPC1 channel in B16F10 cells. (**A**) Triplicate total RNA samples from control and the siRNA-transient receptor potential canonical 1 (TRPC1) transfected cell were analyzed by RT-PCR. GAPDH was used as the standard control. * *p* < 0.05. (**B**) siRNA-TRPC1-transfected B16F10 cells were stained with Cal-520 AM to observe the intracellular calcium ions levels through fluorescence intensity levels. Data are presented as mean ± standard deviation (SD) of at least three independent experiments. ** *p* < 0.01. Treatment or transfection related abbreviations: S1 = sample 1; S2 = sample 2; siRNA-TRPC1 = transfection of siRNA-TRPC1.

To investigate which channels were involved in the CaSR-induced Ca^2+^ influx in B16F10 cells, we analyzed the gene expressions of various ion channels including TRPC1/2/3/4 and 6. Of interest, TRPC1 was expressed in the B16F10 cells and was downregulated with siRNA-TRPC1 treatment (Figure 5A). We also observed mRNA expression of TRPC6; however, these levels were very low and negligible (data are not shown). To confirm the correlation of CaSR-induced Ca^2+^ with TRPC1, we conducted two separate experiments. First, B16F10 cells were transfected with si-RNA-PRP4, which resulted in the inhibition of ARRB1-mediated desensitization of CaSR and increased the influx of Ca^2+^ (as previously shown in Figure 4A). Then, B16F10 cells were transfected with siRNA-TRPC1, which dramatically reduced the influx of Ca^2+^ (Figure 5B), although PRP4 was blocked and CaSR was activated. These findings suggest that PRP4-regulated CaSR-induced Ca^2+^ influx occurs through the TRPC1 channel.

### 2.5. PRP4 Changes the Morphology of B16F10 by Regulating the AC–cAMP–RhoA Pathway

**Figure 6:**
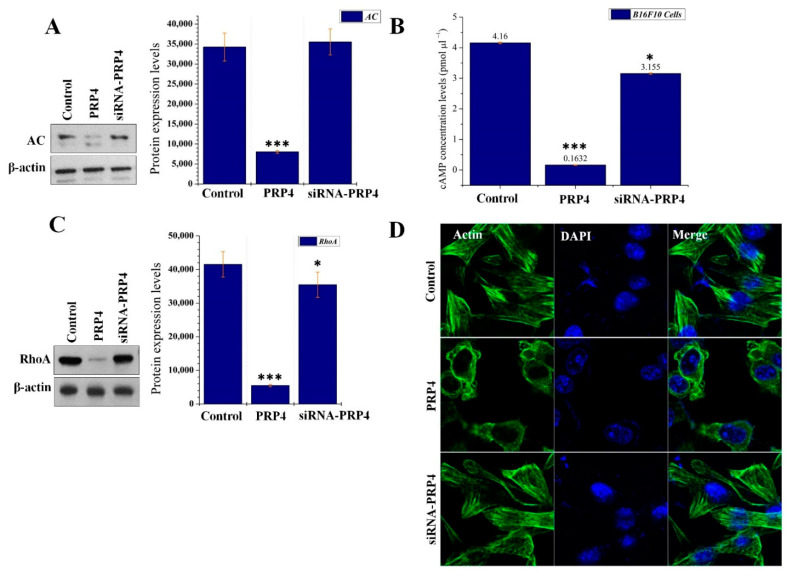
PRP4 alters the morphology of B16F10 cells. (**A,C**) Solubilized proteins (20 μg) from PRP4 and siRNA-PRP4-transfected cultured cells were subjected to Western blot analysis using the corresponding antibodies for detection of respective adenylyl cyclase and RhoA proteins. The blot was simultaneously incubated with an anti-proteins antibody to show that each electrophoretic lane was loaded with the same amount of protein. β-actin was used as a standard. * *p* < 0.05, *** *p* < 0.001. (**B**) Corresponding cells were observed for cAMP levels using a cAMP assay kit, and plated cells were measured in a microplate reader (OD 450 nm). * *p* < 0.05, *** *p* < 0.001. (**D**) PRP4 and siRNA-PRP4 transfected B16F10 cells were stained with Alexa Fluor™ 488 Phalloidin to observe the structure of actin stress fiber formation. Nuclei were counterstained with DAPI, and cells were imaged using a confocal microscope (Carl Zeiss).

Ca^2+^ either directly or indirectly (via calmodulin) regulates cAMP synthesis [48]. In connection with the effect of PRP4 overexpression upon intracellular Ca^2+^, to analyze the regulatory effect of PRP4 on AC and cAMP was of interest in this study. B16F10 were transfected with PRP4 overexpression plasmid and total cell lysates were analyzed by Western blot. It was observed that PRP4 overexpression significantly downregulated the expression of AC, whereas inhibition of PRP4 through siRNA restored the expression of AC (Figure 6A). Next, we analyzed the effect of PRP4 overexpression on the synthesis of cAMP. In line with the inhibition of AC by PRP4 overexpression, cAMP synthesis was also blocked by PRP4 and subsequently restored after siRNA-mediated knockdown of PRP4 (Figure 6B). Multiple previous studies have shown that cAMP regulates RhoA and leads to morphological changes in the cells [49]. Previously, we have reported that PRP4 overexpression induced actin filament redistribution and modified cell morphology from an aggregated, flattened shape to a round shape through inhibition of RhoA activity. To evaluate whether the regulatory effect of PRP4 upon AC and cAMP affected RhoA, we conducted Western blot analysis using the same cell lysates as used in Figure 6A. We observed that PRP4 downregulated the activity of RhoA, and the action of PRP4 was reversed after the utilization of siRNA-PRP4 (Figure 6C). Due to the PRP4 regulatory action on RhoA, we analyzed the actin cytoskeleton of B16F10 cells. This data demonstrated that PRP4 effectively regulated the actin cytoskeleton and morphology of the B16F10 cells, while not affecting the nuclei of the cells (Figure 6D). These findings suggest that PRP4 remodels the morphology of B16F10 by regulating the AC–cAMP–RhoA pathway.

## 3. Discussion

B16F10 cells have been extensively used for studies of melanogenesis and depigmentation [50]. In this study, we were interested to analyze the impact PRP4 has upon melanin production. B16F10 cells were treated with α-MSH followed by PRP4/siRNA-PRP4 transfection. The results demonstrated that PRP4 overexpression blocked the α-MSH-induced melanin production in B16F10 cells. Upon further confirmation, we found that mRNA and protein levels of tyrosine were consistently downregulated by PRP4 overexpression. We suggest that PRP4 decreased the production of melanin in B16F10 cells by downregulating AC–cAMP–MITF–tyrosine signaling pathway, which may promote skin carcinogenesis and drug resistance.

Other supportive mechanisms of PRP4 in promoting skin carcinogenesis and induction of drug resistance were evaluated in this study. PRP4 upregulated the expression of ARRB1, which in turn desensitized CaSR. Upon desensitization, CaSR inhibited the influx of Ca^2+^, which was evident from the observed decreased calmodulin expression. Our results are consistent with previous investigations, which report that Ca^2+^ is deregulated in cancer [51,52,53,54]. To confirm the link of PRP4 with ARRB1-CaSR-calmodulin, we utilized siRNA-PRP4, which silenced PRP4 and inhibited its effect upon ARRB1, CaSR, and calmodulin.

Ca^2+^ influx is induced by CaSR through store-operated channels, which, in many cases, have been identified as TRP family members [23]. We were interested in identifying the channel through which CaSR mediated the Ca^2+^ influx in B16F10 cells. For this purpose, we analyzed the gene expressions of various ion channels including TRPC1/2/3/4 and 6 in B16F10 cells. We noted that only TRPC1 mRNA expression was detectable, whereas TRPC6 presented very low mRNA expression levels. TRPC2/3 and 4 were not present in B16F10 cells. Furthermore, we transfected B16F10 cells with siRNA-PRP4, which resulted in the inhibition of ARRB1-mediated desensitization of CaSR and increased the influx of Ca^2+^. We subsequently transfected B16F10 cells with siRNA-TRPC1, which dramatically reduced the influx of Ca^2+^. Our findings suggest that PRP4 regulated CaSR-induced Ca^2+^ influx occurs through the opening of the TRPC1 channel.

Previously, we have shown that PRP4 induced changes to the cell cytoskeletal architecture and thereby induced EMT. Although PRP4 has been shown to alter cell cytoskeletal architecture in multiple cell lines, we have demonstrated these findings in B16F10 cell lines [44]; where it was observed that PRP4 transfection significantly decreased the expression of AC. We also analyzed the effect of PRP4 overexpression on the synthesis of cAMP. In line with the inhibition of AC by PRP4 overexpression, cAMP synthesis was also inhibited by PRP4. We further analyzed whether PRP4 is involved in the cAMP regulatory action on cell morphology [49]. Through Western blot, we observed that PRP4 downregulated the activity of RhoA, and effectively remodeled the actin cytoskeleton and morphology of the B16F10 cells. These findings indicate that PRP4 remodels the morphology of B16F10 cells by regulating the AC–cAMP–RhoA pathway. These results also serve as an extension to our previous study in which it was reported that PRP4 overexpression inhibited the Rho–ROCKLIMK–cofilin pathway and ultimately induced cofilin dephosphorylation, which resulted in F-actin stabilization and redistribution of cytoplasmic actin [45].

## 4. Materials and Methods

### 4.1. Chemicals and Reagents

Cell lines were purchased from the American Type Culture Collection (Manassas, VA, USA). Dulbecco’s modified Eagle’s medium (DMEM), fetal bovine serum (FBS), and penicillin/streptomycin were obtained from Gibco (Carlsbad, CA, USA). We purchased the PRP4 cDNA clone (Cat# HG10835-M) and ARRB1 cDNA clone (Cat# HG12310-CF) from Sino Biological (North Wales, PA, USA). siRNA-ARRB1 (sc-29741), siRNA-PRP4 (sc-76257), and siRNA-TRPC1 (SC-42664) were purchased from Santa Cruz. Lipofectamine RNAiMAX Transfection Reagent was purchased from Invitrogen. Xfect transfection reagent was purchased from Takara Bio USA, Inc. (Mountain View, CA, USA). Cal-520 AM assay kit (ab171868), 1 mM Ca^2+^ supplemented buffer, and cAMP assay kit (ab65355) were obtained from Abcam. Bradford protein assay kit and electrophoresis reagents were purchased from Bio-Rad Laboratories (Irvine, CA, USA). Antibodies were obtained from Santa Cruz Biotech [PRP4 (sc-130856), β-actin (sc-47778), ARRB1 (sc-53780), Tyrosinase (sc-20035), CaSR (sc-47741), adenylyl cyclase (sc-365350)] and cell signaling technology [calmodulin (cat #4830), RhoA (cat # 2117), anti-mouse IgG-HRP-linked (cat #7076), and anti-rabbit IgG-HRP-linked (cat #7074)]. ECL Prime detection reagent and nitrocellulose membrane were purchased from Amersham (Little Chalfont, Buckinghamshire, UK). Vectashield mounting medium with DAPI (4′,6-diamidino-2-phenylindole) from Vector Laboratories Inc. (Burlingame, CA, USA) was used for nuclei staining. SuperScript III Reverse Transcriptase (Cat #18080093) was obtained from Invitrogen (Carlsbad, CA, USA). Actin-stain™ 488 Phalloidin (A12379) was obtained from ThermoFisher scientific, and α-MSH (M4135) was purchased from Sigma Aldrich. All chemicals and reagents were used according to the manufacturer’s instructions.

### 4.2. Cell Culture and Double Transient Transfections

B16F10 (ATCC CRL-6475) cells were cultured in DMEM containing 1 mM Ca^2+^, supplemented with 10% FBS and 2% penicillin–streptomycin, and were maintained at 37 °C in a humidified atmosphere containing 5% CO_2_.

### 4.3. Plasmid Transfection and Gene Knock Down by siRNA

PRP4 overexpression was achieved by transfecting cells with PRP4 expression plasmids. Briefly, B16F10 cells were cultured at a density of 1 x 10^6^ cells to 50% confluence. Then, the PRP4 plasmid was transfected into the cells using the Xfect Transfection Reagent, whereas siRNA-PRP4 was transfected with RNAiMAX, according to the manufacturer’s instructions. Similarly, siRNA-ARRB1 and siRNA-TRPC1 were also transfected with RNAiMAX. Gene overexpression and downregulation were confirmed by agarose gel electrophoresis and SDS-PAGE.

### 4.4. Reverse Transcription-Polymerase Chain Reaction (RT-PCR)

For cDNA synthesis, total RNA (5 µg) was reverse transcribed using the SuperScript III First-strand synthesis kit as previously described [23]. The synthesized cDNA was incubated with RNase H at 37 °C for 2 h. PCR was performed using 2 μL of cDNA and primers used were PRP4 forward, 5ʹ -AGGGATCGAAGCTGGAAATA-3ʹ and PRP4 reverse, 5ʹ -TGACCTCTGAGTCATCT-GTGG-3ʹ; CaSR forward, 5ʹ-CTGAAGAGAAGGCAACGCTATG-3ʹ and CaSR reverse, 5ʹ- GGGCAACAAAACTCAAGGT -3ʹ; ARRB1 forward, 5ʹ-CGGATGCTTTCTCGTCTC-3ʹ and ARRB1 reverse, 5ʹ-ACCCATCATCATTGTGCC-3ʹ; calmodulin forward 5ʹ-CCCTCTGTCCACACACAAAG-3ʹ and calmodulin reverse, 5ʹ-TTGATGGTGTGCTCAAGTCC-3ʹ, TRPC1 forward 5′-CCTCCTTGTTCTGTTTTCCTTC-3′; and TRPC1 reverse 5′-GTGTCATTGCTTTGCTGTTC-3ʹ; TYR forward 5′- GCTGCAGGAGCCTTCTTTCTC-3′; and TYR reverse 5′-AAGACGCTGCACTGCTGGTCT-3ʹ;, glyceraldehyde-3-phosphate dehydrogenase (GAPDH) forward, 5ʹ-AGGGCTGCTTTTAACTCTGGT-3ʹ and GAPDH reverse, 5ʹ-CCCCACTTGATTTTGGAGGGA-3ʹ. PCR cycles were run as follows: one cycle at 98 °C for 3 min, followed by 30–35 cycles at 95 °C for 30 s, 55 °C for 30 s, and 72 °C for 30 s, with a final extension step at 72 °C for 5 min. The amplified PCR products were analyzed by 2% agarose gel electrophoresis and EcoDye Nucleic Acid Staining Solution (Biofact). Images were subsequently captured using Wise Capture I-1000 software (Daihan Scientific, Seoul, Korea).

### 4.5. Relative qRT-PCR Analysis

Trizol reagent was used to extract total RNA (Life Technologies, Grand Island, NY, USA) according to the manufacturer’s instructions. qRT-PCR was performed in NanoQ (OPTIZEN, Daejeon, Korea) with reagents obtained from TaKaRa Biotechnology Co., Ltd. (Dalian, China). DNA (cDNA) was synthesized as per manufacturer protocol SuperScript^®^ VILO™ cDNA synthesis kit (Life Technologies). PCR cycles were performed with specific primers using a two-step reaction and run using the Eco TM Real-Time PCR system (Illumina, CA, USA). The master mix consisted of 2 x Real-Time PCR Master mix containing SYBR Green I (BIOFACT, Korea) with 100 ng of template DNA and 10 nM of each primer in a final volume of 20 μL. The qPCR cycle was run at 95 °C for 15 min, with concurrent denaturation at 95 °C, annealing and extension at 60 °C for 34 s for 40 cycles. Primers for PRP4, ARRB1, CaSR, and GAPDH were the same as those described for RT-PCR.

### 4.6. F-Actin Staining

Alexa Fluor 488 phalloidin was used for F-actin visualization. Briefly, after removal of the growth medium, cells were washed twice with PBS and fixed with 4% paraformaldehyde for 15 min at room temperature. Cells were permeabilized with 0.2% Triton X-100 for 5 min and washed 2–3 times with PBS. The Alexa Fluor 488 phalloidin stock solution (6.6 µM in methanol) was diluted 1:40 with 1% bovine serum albumin and added to the cells for 50 min at room temperature in the dark. Cells were further washed with PBS 5–6 times and actin cytoskeleton were observed using a ZEISS LSM 800 confocal microscope at 1000× magnification.

### 4.7. Cal-520 AM Assay

B16F10 cells were cultured in DMEM containing 1 mM Ca^2+^ supplemented with 10% fetal bovine serum (Hy Clone, USA) in 5% CO_2_ at 37 °C. Cells were transfected with respective siRNAs and incubated for 6 h in serum-free optimum medium. At 7 h, full serum DMEM media was added to the cells and incubated for a following 17 h. Cells were subsequently loaded with Cal-520 AM dye (10 mM) in 200 μL HHBS buffer with Pluronic F-127 (0.01%) for 30 min at 37 °C. This indicator has high-affinity binding to Ca^2+^ (K_d_ = ¼ 345 nM) and will indicate a significant increase in fluorescence intensity in response to Ca^2+^ binding (>100 fold). Cells were washed twice with HHBS Solution, and images of calcium-dependent fluorescence were obtained using a FITC- ZEISS LSM 800 confocal microscope 492 nm.

### 4.8. cAMP Assay

B16F10 cells were cultured and transfected with PRP4, then collected and lysed, and the levels of cAMP intracellularly were assessed using a cAMP assay kit according to the manufacturer’s protocol. The assay was repeated in triplicate.

### 4.9. Animal Study Protocol

We housed 6 male BALB/c-n mice at a density of two mice per cage, under conditions of constant temperature (22 °C) and a light/dark cycle of 12 h. Our experimental protocol complied with the animal maintenance and use guidelines of Kyungpook National University. We suspended a total of 10^6^ parental or PRP4-transfected B16F106 cells in 150 µL PBS and implanted them subcutaneously into 6-week-old mice at specific left and right sites using an insulin syringe. We recorded tumor volumes weekly using a Vernier caliper and calculated them according to the formula: V = 4/3πW2L (short diameter2 × long diameter [mm3]). We excised the tumors 30–45 days after cells’ postimplantation using scissors.

### 4.10. Measurement of Melanin Content

B16F10 cells were seeded into a 60 mm dish and incubated for 24 h. Next, media was replaced with serum-free optimum media, and the cells were transfected with PRP4, and incubated with 200 nM α-MSH for 24–72 h. After the incubation period, cells were washed with PBS and dissolved in 2 mL 1 N NaOH containing 10% DMSO at 70 °C for 1 h. To this, 200 μL NaOH solution aliquots were added to the 96-well plate, and the absorbance was measured at 405 nm on a spectrophotometric microplate reader (Tecan, Mannedorf, Switzerland).

### 4.11. Western Blot

Cells were collected with a cell scrapper in PBS and centrifuged at 12,000 rpm for 5 min to obtain the cell pellet. The supernatant was discarded, and the cell pellet was resuspended in 200 µL of cell lysis buffer [50 mM Tris, pH 7.4, 0.5% NP40, 0.01% SDS, and protease inhibitor cocktail (Roche, Germany)]. Total cells lysates were then quantified using the Bio-Rad Protein Assay according to the manufacturer’s protocol. Samples (20–40 μg) were prepared in SDS sample buffer containing 60 mM Tris-HCl (pH 6.8), 2% SDS, 10% glycerol, and 5% β-mercaptoethanol, and separated on a 10–12% SDS-PAGE gel and transferred onto a polyvinylidene fluoride (PVDF) membrane (Amersham, Piscataway, NJ, USA). Membranes were blocked with 3% albumin (Gendepot, USA) solution for 2 h at 4 °C. Chemiluminescent signals were developed using a Clarity ECL Western Blotting Substrate (Bio-Rad) according to the manufacturer’s instructions.

### 4.12. Statistical Analysis

All samples and experiments were prepared and run-in triplicate. Data are presented as mean ± standard deviation (SD). A Student t-test was used to evaluate differences between groups and *p*-values < 0.05 were considered statistically significant.

## 5. Conclusions

Our study demonstrates that PRP4 inhibits the production of melanin in B16F10 cells, which may promote skin carcinogenesis and drug resistance. The two other supporting mechanisms for cancer promotion and drug resistance in B16F10 cells include PRP4-induced inhibition of Ca^2+^ influx through CaSR desensitization and remodeling of the cell actin cytoskeleton by the downregulation of the AC–cAMP–RhoA signaling pathway (Graphical abstract).

## Data Availability

The data that support the findings of this study are available on request from the corresponding author.

## Abbreviations

PRP4	Pre-mRNA processing factor 4B
EMT	Epithelial-mesenchymal transition
AC	Adenylyl cyclase
CaSR	Calcium-sensing receptor
RhoA	Ras homolog family member A
TRP-1	Tyrosinase-related protein
MITF	Microphthalmia-associated transcription factor
CREB	cAMP response element-binding protein
ERK	Extracellular-regulated kinase
α-MSH	Alpha-melanocyte stimulating hormone
GPCR	G-protein-coupled receptor
GRKs	G protein-coupled receptor kinase
TRP	Transient receptor potential
TRPC1	Transient receptor potential canonical 1
cAMP	Cyclic adenosine 3′,5′-monophosphate
MC1R	Melanocortin 1 receptor
